# Ficolins and the Recognition of Pathogenic Microorganisms: An Overview of the Innate Immune Response and Contribution of Single Nucleotide Polymorphisms

**DOI:** 10.1155/2019/3205072

**Published:** 2019-02-05

**Authors:** Stefan Bidula, Darren W. Sexton, Silke Schelenz

**Affiliations:** ^1^School of Pharmacy, University of East Anglia, Norwich Research Park, Norwich NR4 7TJ, UK; ^2^School of Pharmacy and Biomolecular Science, Liverpool John Moores University, Byrom Street, Liverpool, L3 3AF, UK; ^3^Department of Microbiology, Royal Brompton Hospital, Sydney Street, London SW3 6NP, UK

## Abstract

Ficolins are innate pattern recognition receptors (PRR) and play integral roles within the innate immune response to numerous pathogens throughout the circulation, as well as within organs. Pathogens are primarily removed by direct opsonisation following the recognition of cell surface carbohydrates and other immunostimulatory molecules or via the activation of the lectin complement pathway, which results in the deposition of C3b and the recruitment of phagocytes. In recent years, there have been a number of studies implicating ficolins in the recognition and removal of numerous bacterial, viral, fungal, and parasitic pathogens. Moreover, there has been expanding evidence highlighting that mutations within these key immune proteins, or the possession of particular haplotypes, enhance susceptibility to colonization by pathogens and dysfunctional immune responses. This review will therefore encompass previous knowledge on the role of ficolins in the recognition of bacterial and viral pathogens, while acknowledging the recent advances in the immune response to fungal and parasitic infections. Additionally, we will explore the various genetic susceptibility factors that predispose individuals to infection.

## 1. Introduction

**Figure 1:**
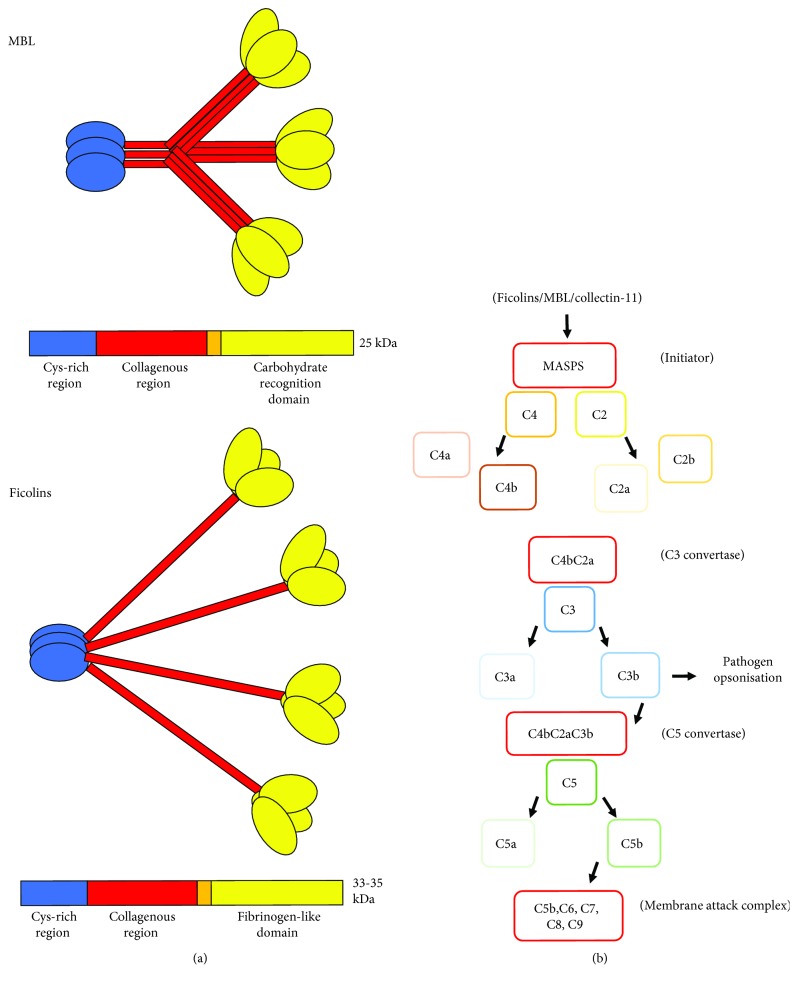
Ficolin structure and the lectin complement pathway. (a) MBL is composed of a cysteine-rich region, a MASP-interacting collagenous region, and a pathogen-binding carbohydrate recognition domain. Ficolins have structural similarity to MBL, albeit the carbohydrate recognition domain is replaced by a fibrinogen-like domain. (b) There are three main pathways of complement activation: the classical, lectin, and alternative pathways. Ficolins interact with MASPs to cleave C4 and C2 to form the C3 convertase C4bC2a. This results in the cleavage of C3 into C3a and C3b. C3b then functions as an opsonin or enters the alternative pathway forming an amplification loop. Each pathway can also result in the formation of the membrane attack complex following the cleavage of C5 by the C5 convertases C4bC2aC3b or C3BbC3b and subsequent association with C6, C7, C8, and C9.

Ficolins are innate pattern recognition receptors (PRRs) similar to the collectin, the mannose-binding lectin (MBL), and the surfactant proteins (SP). Like the collectins, ficolins consist of an N-terminal rich in cysteine residues, a collagen-like domain composed of glycine-X-Y repeats, and a neck region. However, in the ficolins, the carbohydrate-recognition domain (CRD) of the collectins is replaced by a C-terminal fibrinogen-like domain (FBG; Figure 1(a)). In their native form, ficolin monomers assemble to form trimers via their collagen-like domains, before further assembling into oligomeric bouquet-like structures of between 4 and 6 trimers. In humans, there are three ficolins termed M-, L-, and H-ficolin (also referred to as ficolin-1, ficolin-2, and ficolin-3) whereas rodents only possess two, termed ficolin-A and -B, which are the orthologues of human L- and M-ficolin, respectively. The H-ficolin gene is present in rodents as a pseudogene [1].

Ficolins function within innate immunity via the recognition of pathogen-associated molecular patterns (PAMPs) on microbial pathogens. Binding to acetylated polysaccharides on microbial pathogens, in particular *N*-acetylglucosamine (GlcNAc) and *N*-acetylgalactosamine (GalNAc), is a common characteristic shared amongst the ficolins. However, ficolins have also been observed to recognise specific microbial patterns such as sialic acid, lipopolysaccharides, bacterial peptidoglycan, and fungal 1,3-*β*-D-glucan [2–6]. Following the recognition of cell surface structures during infection, ficolins can function as opsonins, potentiating the functions of leukocytes and the lung epithelium [7–9].

To date, all of the human and rodent ficolins have been observed to activate the lectin-complement pathway (Figures 1(b) and 1(c)) following the association with MBL-associated serine proteases (MASPs), a characteristic shared with MBL and the recently discovered collectin-11 (CL-11) [10–17]. Ficolins associate with three serine proteases, MASP-1, MASP-2, and MASP-3, in addition to two nonenzymatic fragments MAp19 and MAp44 [18–20]. Ficolin-MASP complexes can then cleave C4 and C2 to form the C3 convertase C4bC2a [11, 21]. Following the deposition of C3b on C4bC2a, C2a gains C5 convertase activity leading to the production of C5a and C5b. C3b itself acts as a marker to facilitate phagocytosis, and C5b initiates the formation of the membrane attack complex (MAC) in collaboration with C6, C7, C8, and C9, which directly lyses pathogens [22].

In this review, we describe updates on the opsonic activity of human and rodent ficolins and explore their role within innate immune responses against pathogens. Moreover, we briefly discuss the effects of single nucleotide polymorphisms on pathogen susceptibility.

## 2. The Ligand-Binding Properties of Ficolin Fibrinogen-like (FBG) Domains

The FBG of ficolins is composed of a number of different binding sites that can work synergistically or alone in a complex interaction that allow ficolins to distinguish nonself structures from self. This allows ficolins to play an integral role in the opsonisation of various pathogens whereby they can recognise a vast number of ligands on the microbial cell-surface.

In recent years, it has been discovered that the binding range of M-ficolin is a lot broader than was first anticipated, and the tethering of M-ficolin to the leukocyte surface is due to the recognition of sialic acid by the FBG, in particular 9-*O*-acetylated sialic acid [2, 23–25]. Moreover, like other ficolins, M-ficolin can bind to a vast range of acetylated moieties including *N*-acetylglucosamine (GlcNAc), *N-*acetylgalactosamine (GalNAc), *N*-acetyllactosamine (LacNAc), *N*-acetylcysteine (CysNAc), and acetylated human serum albumin [10, 23, 26]. A glycan array further advanced our knowledge on the recognition properties of M-ficolin and highlighted two novel ligands in the form of gangliosides and sialylated biantennary N-linked type glucans [25]. M-ficolin has also recently been implicated in neutrophil responses, including cell polarization, adhesion, aggregation, and complement activation, following interaction with the membrane sialoprotein CD43 [27]. The M-ficolin recognition domain has been studied in great detail. The structure is similar to the other ficolins; however, within a predicted ligand-binding site, the peptide bond between Asp282 and Cys283 is in a normal *trans* conformation, compared to the *cis* conformation exhibited by the other ficolins [28]. The difference between active and nonactive function was suggested to be due to a *cis*-*trans* isomerization of the Asp282 and Cys283 peptide bond [29], with an acidic environment gearing M-ficolin towards the nonfunctional *trans* conformation. Using various histidine mutants, the protonation of His284 was found to be associated with the *trans* to *cis* change to a functional conformation and the ability to regulate GlcNAc binding [30, 31].

L-ficolin is the best characterised of all of the ficolins and binds to a wide range of antigens, thus allowing L-ficolin to recognise an array of microorganisms. L-ficolin shares a common binding specificity to GlcNAc and GalNAc but also binds to a wider range of structures such as lipoteichoic acid (LTA), 1,3-*β*-D-glucan, N-glycans, hemagglutinin (HA), and neuraminidase [5, 6, 32–34]. Due to the large recognition spectrum of L-ficolin, the incorporation of sites other than the S1 binding site, termed S2-S4, is of great importance. The S1 site, important in the recognition of GlcNAc for the other ficolins, contains a phenylalanine residue in place of a GlcNAc stabilising tyrosine in L-ficolin and is less involved [35]. Alternatively, GlcNAc, CysNAc, and neutral galactose were found in the S2 site; various N-acetylated structures in the S3 site and a cooperation of the S3 and S4 sites were involved in the recognition of 1,3-*β*-D-glucan, altogether producing a unique recognition surface for the recognition of pathogens [35].

H-ficolin shares common binding specificities with the other ficolins, namely, the recognition of the acetylated polysaccharides GlcNAc and GalNAc, but, additionally D-fucose and galactose [3, 35]. Structurally, H-ficolin shares characteristics with L-ficolin such as the common *cis* conformation of the Asp282 and Cys283 peptide bond [35, 36]. Garlatti et al. [35] characterised the binding of H-ficolin and elucidated the S1 site which was involved in binding to both D-fucose and galactose. As in the other ficolins, this site lies within the vicinity of the Ca^2+^-binding site and is homologous to the GlcNAc-binding site in tachylectin 5A, involving Cys235, Tyr236, Tyr254, and Val264 residues [35]. Zacho et al. [37] further characterised the binding profile of H-ficolin reporting binding to acetylsalicylic acid, *N*-acetylglycine (GlyNAc), and CysNAc and reporting the Ca^2+^ dependence of H-ficolin binding. However, the sites involved in this recognition need to be investigated further.

## 3. The Role of M-Ficolin in Immunity to Pathogenic Microorganisms

### 3.1. Bacteria

As a consequence of its recognition spectrum (Table 1), it has been suggested that M-ficolin could be involved in immunity against bacterial infection. Dose-dependent binding of serum M-ficolin has been observed to capsular *Streptococcus agalactiae* serotype VI, which presents sialic acid as the terminal side-chain residues of the capsular polysaccharides, but does not bind to the noncapsulated strain B848/64 [38]. This concentration-dependent binding was inhibited following the addition of GlcNAc or treatment of bacteria with sialidase. Recombinant M-ficolin also exhibited the same binding preferences and activated complement only on serotype VI streptococci [38]. The same group reported that M-ficolin was unable to bind to either the capsulated or noncapsulated strains of *Staphylococcus aureus*, contradicting a previous observation by Liu et al. [26]. To further characterize M-ficolin binding, more than 100 different strains of *Streptococcus pneumoniae* and *Streptococcus mitis* were screened for M-ficolin binding. M-ficolin was only observed to bind to three strains: the pneumococcal serotypes 19B and 19C and a single *S. mitis* strain [39]. This binding exhibited the common characteristic of GlcNAc inhibition and in conjunction with MASP-2, mediated the cleavage of C4. Kjaer et al. [39] postulated that binding to these pneumococcal strains was via an *N*-acetylmannosamine residue linked by glycoside linkage only present in serotypes 19B and 19C. In addition, M-ficolin can function as an opsonin and enhance the action of phagocytes, as phagocytosis of *Escherichia coli* by U937 cells was observed to be inhibited by antibodies against M-ficolin [23].

Bartlomiejczyk and colleagues also demonstrated M-ficolin binding to *Mycobacterium bovis* and *M. tuberculosis*, although this binding had little effect on biological responses [40].

### 3.2. Viruses

Verma et al. [41] identified that M-ficolin could recognise and inhibit the infectivity of the Phil82 and PR-8 strains of the influenza A virus (IAV) to levels comparable to L-ficolin. H-ficolin in this study was also capable of inhibiting the infectivity of the pandemic Cal09 H1N1 strain. Furthermore, M-ficolin was also observed to interact with acute phase proteins which could potentiate the immune response against pathogens. Using surface plasmon resonance spectroscopy and electron microscopy, the interaction of M-ficolin with the long pentraxin, pentraxin 3 (PTX3), was investigated. M-ficolin was shown to bind PTX3 in a Ca^2+^-dependent manner in an interaction inhibited by GlcNAc. The M-ficolin-PTX3 interaction was attributed to sialic acid, and the activation of the lectin-complement pathway was observed [42]. Functionally, the M-ficolin-PTX3 interaction has been observed to decrease the infectivity of the PR-8 and Phil82 strains of IAV [41]. Conversely, interactions of M-ficolin with the mucin-like domain of the Zaire Ebola virus glycoprotein results in enhanced infectivity of host cells [43].

### 3.3. Fungi

Until recently, there had been no reports of M-ficolin binding to *A. fumigatus*; however, Jensen et al. [44] identified that M-ficolin can interact with chitin and *β*-1,3-glucans, contributing to complement activation and potentiation of IL-8 from a lung epithelial cell line. The ability of M-ficolin to recognise such key components of the fungal cell wall, as well as its production by peripheral blood leukocytes and type II alveolar cells, is suggestive that its importance in antifungal immunity will be further unveiled in time.

### 3.4. Parasites

There has been a paucity of information regarding the role of ficolins in immunity to parasites, but due to its recognition spectrum, it would seem likely that M-ficolin would interact with parasites and is an area that needs to be explored.

## 4. The Role of L-Ficolin in Immunity to Pathogenic Microorganisms

### 4.1. Bacteria

L-ficolin is undoubtedly the most widely investigated ficolin, and studies have identified important roles within infection and immunity. L-ficolin was first observed to enhance the opsonophagocytosis of *Salmonella typhimurium* leading to complement activation [7]. It should be noted that in this study, L-ficolin was demonstrated to bind to the *S. typhimurium* Ra strain lacking LPS *O*-specific polysaccharide but not with the LPS smooth-type strain. L-ficolin recognises LTA expressed by a range of staphylococcal and streptococcal strains, including *S. aureus* serotypes 1, 8, 9, 11, and 12 and *S. pneumoniae* serotypes 11A, 11D, 11F, 20, 35A, and 35C, subsequently leading to activation of the lectin pathway on some serotypes [5, 39]. This binding could be due to the recognition of the pneumococcal surface virulence factors of the choline-binding protein (Cbp) family by the FBG domain [45]. Recent evidence has been provided which demonstrates that L-ficolin recognises O-acetylated epitopes on pneumococcal serotype 11A and contributes to reduced invasiveness [46]. Further delineation of the binding specificity led to the observation that L-ficolin could bind to the PCho residue in teichoic acid [47]. An important study by Ali et al. additionally provided evidence that L-ficolin can activate the lectin pathway of complement via binding pneumolysin, a major toxin of *S. pneumoniae* [48].

Group B streptococci (GBS), in particular capsular polysaccharide (CPS) from serotypes Ib, III, V, VI, and VIII, is also avidly recognised by L-ficolin, leading to a significant increase in opsonophagocytosis and C3b deposition via the lectin pathway working synergistically with the alternative complement pathway [49, 50]. The binding of L-ficolin to GBS is suggested to be irrespective of the amount of LTA or group B-specific polysaccharide (GBPS) content but shows a directly proportional decrease in binding following the removal of N-acetylneuraminic acid (NeuNAc) [51]. L-ficolin and serotype-specific IgG from cord serum have been observed to increase opsonophagocytic killing of serotype III and V [50]. However, recognition of GBS by L-ficolin is a result currently under debate as another group reported no binding to any serotypes investigated [38]. L-ficolin also binds to *Enterococcus faecalis*, *Leptospira biflexa*, *Listeria monocytogenes*, *Pasteurella pneumotropica*, and enteropathogenic or enteroaggregative *E. coli*; however, the functional consequences of these interactions are not fully elucidated [52, 53]. Recent evidence is indicative that L-ficolin may play an important role in immunity against enteroaggregative *E. coli* via activation of the lectin-complement pathway [54]. Moreover, the interaction of L-ficolin with C-reactive protein (CRP) significantly enhanced the complement deposition on *P. aeruginosa* [55].

L-ficolin has arisen as an important defence molecule within the liver in particular, whereby lower L-ficolin levels are correlated with an increased incidence of bacterial infections and disease severity during sepsis [56–58].

Additionally, L-ficolin has been observed to bind to mycobacteria. L-ficolin recognises *Mycobacterium bovis* BCG, leading to complement activation and significant C3b deposition [59]. In this case, C3b deposition could be involved in the virulence of *M. bovis* BCG to allow entry into macrophages where they reside. Moreover, Luo and colleagues identified that L-ficolin could bind more effectively to the virulent *M. tuberculosis* strain H37Rv, comparatively to nonvirulent *M. bovis* BCG and *M. smegmatis* [60]. Notably, an insufficiency of L-ficolin in humans was attributed to enhanced susceptibility to infection with TB [60].

### 4.2. Viruses

A role for L-ficolin in viral defence is now also starting to emerge. Liu et al. [33] found that L-ficolin in patients with hepatitis C virus (HCV) was elevated and was able to bind to N-glycans of the envelope glycoproteins E1 and E2. This interaction further led to the activation of the lectin pathway. Recent evidence has been provided which displays the ability of L-ficolin to directly inhibit HCV entry into cells and demonstrated that apolipoprotein E3 (ApoE3) blocks this effect, mediating immune escape [61, 62]. Recent evidence has indicated that L-ficolin concentrations are elevated in the serum of chronic hepatitis B patients also [63]. Additionally, L-ficolin has been observed to bind to HA and neuraminidase via their FBG and has been shown to have an inhibitory effect on the invasion of kidney cells by IAV *in vitro* [34]. Using mice deficient of the mouse orthologue of L-ficolin, ficolin-A, it was also observed that these mouse demonstrated a greatly decreased survival rate in comparison to WT. However, reconstitution of L-ficolin into a ficolin-A knockout mouse could significantly reduce mortality. Chimeric lectins whereby part of the L-ficolin collagen-like domain was added in place of MBLs have proven beneficial in defence against both the IAV and the Ebola virus [64, 65].

### 4.3. Fungi

L-ficolin has been observed to bind to the pathogenic fungus, *Aspergillus fumigatus*, leading to lectin-complement pathway activation [66]. Complement activation can be further potentiated by an L-ficolin-PTX3 complex. This group also highlighted that the classical and lectin-complement pathways can complement each other, with the classical pathway the preferred method of initiation but the lectin pathway capable of initiating complement in the absence of anti-*Aspergillus* antibodies [67]. Additionally, we showed that the recognition of *A. fumigatus* by the L-ficolin FBG also enhances the association of *A. fumigatus* to the A549 type II epithelial cell line, human primary neutrophils, and monocyte-derived macrophages (MDM) [9, 68]. This interaction was observed to enhance fungal killing and modulate the inflammatory cytokine response, leading to increased production of IL-8 by epithelial cells, while conversely decreasing the production of IL-1*β*, IL-6, IL-8, and TNF-*α* from neutrophils and MDM *in vitro*. We have since observed the presence of L-ficolin in the bronchoalveolar lavage fluid of lung transplant patients with fungal lung infections, hinting at an important role in antifungal defence [68]. Recently, Genster et al. have highlighted that the absence of both the rodent orthologues ficolin-A and ficolin-B sensitised mice to *A. fumigatus* infections [69]. However, the absence of either one alone was not sufficient to enhance susceptibility. Furthermore, very little is understood about the recognition of other pathogenic fungi by L-ficolin, and this could be an important area to investigate, although it has been reported that there is no association observed between levels of any ficolin and intra-abdominal *Candida albicans* infection [70].

### 4.4. Parasites

Parasitic binding is also a characteristic of L-ficolin. L-ficolin has been observed to bind to glycosylated proteins on the cell surface of *T. cruzi* and recognise *Giardia intestinalis*, leading to complement activation [71, 72]. Recent data has shown that *T. cruzi* is also able to manipulate L-ficolin as a virulence factor. *T. cruzi* calreticulin can bind L-ficolin directly and in doing so is observed to inhibit lectin pathway activation in a dose-dependent manner [73].

## 5. The Role of H-Ficolin in Immunity to Pathogenic Microorganisms

### 5.1. Bacteria

Early work from Sugimoto et al. [3] characterised the ability of H-ficolin to induce agglutination of human erythrocytes by recognition of lipopolysaccharides (LPS) from *S. typhimurium*, *Salmonella minnesota*, and *E. coli* O111 coated on their surface. Binding to bacteria has proven to be restricted to only a few species, the most characterised of these is binding to PSA, a polysaccharide of *Aerococcus viridans*, which is now often used as a control for H-ficolin binding and complement activation [12, 74, 75]. However, even H-ficolin recognition of *A. viridians* and *E. coli* is strain specific as binding to the strains *A. viridans* Ring 44 and *E. coli* 74285 was not observed [75]. Recent work by Swierzko et al. [4] increased our current knowledge of H-ficolin-bacteria interactions. They showed that LPS from only four strains of *Hafnia alvei* was recognised by H-ficolin, in particular via their O-specific polysaccharides, leading to C4b deposition in a calcium and magnesium-dependent manner. The interaction between H-ficolin and *H. alvei* has since been investigated further, whereby H-ficolin can augment phagocytosis and promote bacterial killing [76]. In stark contrast, H-ficolin has not been observed to recognise any *S. pneumoniae*, *S. agalacticae*, *S. mitis*, or *S. aureus* strains [38, 39, 75]. In addition, H-ficolin does not bind to other bacteria such as *L. monocytogenes*, *Pseudomonas aeruginosa*, and *Klebsiella pneumoniae* [52]. Recently, H-ficolin binding has been observed to *Pasteurella pneumotropica* and enteropathogenic and enteroaggregative *E. coli* [53]. Bartlomiejczyk and colleagues demonstrated that H-ficolin could bind to *M.* tuberculosis, *M. bovis*, and *M. kansasii*, with binding to the former resulting in bacterial agglutination and enhanced phagocytosis [40]. The mycobacterial antigen Ag85 has arisen as a novel antigen for the ficolins, in particular H-ficolin, where it might influence the interaction of *Mycobacterium* with the extracellular matrix [77].

### 5.2. Viruses

Observations regarding the role of H-ficolin in the defence against viruses are encouraging. Recent studies have exhibited the ability of recombinant H-ficolin, H-ficolin from human serum, and from bronchoalveolar lavage to bind to IAV, the mouse-adapted PR-8H1N1 and a pandemic H1N1 strain [41]. Following recognition, a decrease in the ability of IAV to cause infection *in vitro* was observed. The role of sialic acid in these mechanisms was suggested to be important, as following sialidase treatments and removal of the sialic acid residues decorating H-ficolin, inhibition of IAV was abolished [41]. In addition, H-ficolin was capable of activating the lectin pathway on a surface coated with IAV. As observed for M- and L-ficolin, H-ficolin has the ability to interact with PTX3 in a dose-dependent manner, although it exhibits the weakest binding of the three [66]. This H-ficolin-PTX3 interaction, as for M-ficolin, exhibited the ability to inhibit HA activity and infectivity of IAV [41]. A more recent study further consolidated these earlier observations by demonstrating that H-ficolin can inhibit the replication of pandemic IAV by enhancing uptake and dampening TNF-*α* response in monocytes [78].

### 5.3. Fungi

Similar to L-ficolin, H-ficolin has been implicated in the recognition of *A. fumigatus*. This interaction led to the activation of the lectin pathway of complement, enhanced association of conidia with A549 epithelial cells, and increased IL-8 production [79]. Moreover, H-ficolin has been shown to be recruited to the lungs during inflammation, and we have observed increased concentrations of H-ficolin in the lungs of transplant patients with proven or probable *A. fumigatus* infection [79, 80].

### 5.4. Parasites

As for L-ficolin, H-ficolin has also recently been observed to bind to the parasites *T. cruzi* and *G. intestinalis* and play a role in complement activation [71, 72]. Depletion of ficolins and MBL led to a 70% decrease in C3b and C4b deposition on *T. cruzi* [81].

## 6. The Role of Rodent Ficolin-A and Ficolin-B in Immunity to Pathogenic Microorganisms

### 6.1. Bacteria

Until recently, the recognition spectrum of ficolin-A with microorganisms was relatively unknown. However, there has been much progress in characterising the interaction of ficolin-A with pathogens. Hummelshøj et al. [52] have greatly expanded current knowledge on the pathogen specificity. They showed ficolin-A to recognise a plethora of microorganisms including pathogenic gram-positive and -negative bacteria such as *S. aureus* and the pathogenic *E. coli* strain O157:H7. They also exhibited the ability of ficolin-A to bind LPS from *E. coli* and *P. aeruginosa*. However, recognition of LPS did not equate to protection or inflammatory modulation in an *in vivo* model of LPS-induced systemic inflammation [82].

Ficolin-A has been observed to partake in the activation of the lectin-complement pathway via the association with MASPs. Further interactions with fibrinogen and thrombin have been observed to potentiate the activation of complement on *S. aureus* [83]. It has also proven to be an essential activator of the lectin-complement pathway in the defence against *S. pneumoniae.* Ficolin-A and -B opsonisation of *S. pneumoniae* leads to complement deposition in the presence of ficolin-A and only weakly in its absence [15]. The role of the lectin-complement pathway in pneumococcal defence has been shown to be important [84].

### 6.2. Viruses

To date, there have been very few studies implicating ficolin-A or -B in viral recognition. Pan et al. [34] however did demonstrate that reconstitution of a ficolin-A KO mouse with either L-ficolin or ficolin-A could ameliorate the effects of IAV and protect against mortality and inflammation.

### 6.3. Fungi

We have further characterised the interactions of ficolin-A with *Aspergillus* spp. *in vitro*. Ficolin-A was observed to recognise *A. fumigatus*, *A. flavus*, *A. niger*, and *A. terreus* with its FBG in a calcium-independent manner. In addition to this, recognition of the most pathogenic species, *A. fumigatus*, was greatly increased in acidic conditions, an interaction which led to enhanced association with the lung epithelium and immobilisation of the fungus [9]. Ficolin-A has also been observed to enhance the phagocytosis of *A. fumigatus* and *A. flavus* by RAW macrophages (unpublished observation), neutrophils, and human macrophages [85]. As previously mentioned, Genster et al. have elucidated an important role for ficolin-A *in vivo* against *A. fumigatus* infection [69]. In addition to this, we have also characterised the binding to the pathogenic yeast *Cryptococcus neoformans.* This interaction shared many of the characteristics of binding to *Aspergillus* such as pH dependence and Ca^2+^ independence, in addition to increased epithelial cell adherence [86].

We also recently elucidated a role of the lectin pathway of complement in the defence against *A. fumigatus in vitro*. In the absence of ficolin-A, complement was activated, but in the absence of MBL-A and -C, no complement activation could be observed. MBL-C but not MBL-A was able to bind to *A. fumigatus*; therefore, we postulated that MBL-C could be the activator of the lectin pathway in the defence against *Aspergillus* [9]. Indeed, fungal clearance in ficolin-A knockout mice appeared to be independent of complement activation [69].

Ficolin-A has also exhibited both pro- and anti-inflammatory potential. Recent evidence suggests that ficolin-A may be capable of binding to LPS- and inhibiting TLR-4-mediated inflammation on mast cells [87]. We have previously observed that ficolin-A is capable of enhancing IL-8 secretion from A549 cells challenged with *A. fumigatus* and others have attributed decreased cytokine production to be the cause of higher fungal burdens in knockout mice [9, 69].

### 6.4. Parasites

Akin to its human orthologue, ficolin-A is also important in the defence against parasites. In an *in vivo* mouse model, ficolin-A was shown to enhance the immunoprotective activity of the 19 kDa fragment of merozoite surface protein-1 of *Plasmodium berghei* which led to a reduction in invasion and an increase in mouse survival [88, 89]. Conversely, upregulated transcripts of ficolin-A were observed in macrophages obtained from mice with tropical pulmonary eosinophilia [90].

The current knowledge on the role of ficolin-B in the recognition and defence against pathogens is largely unkown, but Endo et al. [15] have suggested a synergistic role of ficolin-A and -B in the defence against *S. pneumoniae.* [91].

## 7. Ficolin Single Nucleotide Polymorphisms and Haplotypes Contribute to Pathophysiology

Single nucleotide polymorphisms (SNPs) can have significant effects on the susceptibility to various infections by altering the function or concentration of ficolins found within serum or organs. Early research in the Garred laboratory indicated that there were large ethnic differences in the distribution of SNPs [92]. Moreover, the number of SNPs found in *FCN3* was very low in comparison to *FCN1* and *FCN2*. Many SNPs were predicted to have a major effect on the function of their respective proteins, with one in *FCN3* for example, completely disrupting the FBG. Since then, there has been much evidence implicating the effects of SNPs within ficolins (Table 2).

### 7.1. SNPs in *FCN1*

To date, there have been very few polymorphisms identified in the *FCN1* gene, but polymorphisms and haplotypes have been identified which are linked to antibacterial immunity.

Polymorphisms in *FCN1* have been directly associated with M-ficolin levels, with the +7895T>C mutation resulting in an inability to produce M-ficolin and a mutation at -144C>A resulting in significantly increased levels [93]. The same group identified two other nonsynonymous mutations, one at position +6658G>A and the other at +7959A>G that were associated with low M-ficolin levels, poor ligand-binding capacity, and low binding to group B streptococcus [93]. These observations were made using HEK293 cells transfected with plasmids for the various mutated M-ficolin receptors, albeit computational predictions were made that suggested that these SNPs would be potentially damaging in patients.

Cystic fibrosis patients heterozygous or homozygous for mutant alleles for two SNPs in *FCN1* at position 1981G>A and +7918G>A were more susceptible to earlier colonization by *P. aeruginosa* [94]. Moreover, patients heterozygous for two further SNPs in *FCN2* were also more susceptible to earlier onset of colonization [94].

Boldt and colleagues [95] were the first to implicate *FCN1* SNPs as a risk factor for mycobacterial infections. They identified a combination of SNPs (*FCN1*^∗^-542A-144C) to have an additive protective effect against *M. leprae* infection, albeit there was a negative association of the *FCN1*^∗^3A haplotype with lepromatous leprosy. Genotyping of Danish individuals with known M-ficolin levels highlighted that the *FCN1*^∗^3A haplotype resulted in higher than average M-ficolin levels, which could be an explanation for enhanced susceptibility.

### 7.2. SNPs in *FCN2*

L-ficolin polymorphisms have been the most widely reported, with polymorphisms in the *FCN2* gene contributing to susceptibility to numerous bacterial pathogens and some viruses.

Cedzynski and colleagues initially reported four polymorphisms that were linked to extremes in L-ficolin concentrations in a cohort of Polish children suffering from recurrent respiratory infections [96]. Low L-ficolin levels were associated with variant alleles for -64A>C and +6424G>T but normal alleles for -4A>G and +6359C>T. Conversely, high L-ficolin levels were associated with variant alleles of -4A>G and +6359C>T. *FCN2* polymorphisms were not identified as major risk factors for community acquired pneumonia, albeit there was an association between the +6424G>T polymorphism and patient colonization with *Coxiella burnetii* [97].

The *FCN2* exon 8 +6359 C>T polymorphism has arisen as an important SNP involved in susceptibility to bacterial infections, especially following organ transplants [98, 99]. Wan and colleagues [98] correlated the SNP with increased incidence of bacteraemia in kidney transplants, whereas de Rooij et al. [99] demonstrated an increased incidence of significant bacterial infections and mortality in liver transplant patients. Moreover, the negative effects were cumulative if patients also had SNPs in the lectin pathway components, MBL-2 and MASP-2 [99].

The +6359C>T SNP was also reported as a significant risk factor in patients on continuous ambulatory peritoneal dialysis with a history of staphylococcal peritonitis [100], potentially due to decreased ability to bind to staphylococci.

B acute lymphoblastic leukemia patients were observed to be at a greater risk of bacterial infections and present prolonged episodes of febrile neutropenia if they possessed a medium-/high-risk haplotype for *FCN2* of GGATG, GGACG, or AGACG (all haplotypes composed of -986/-602/-4/+6359/+6424) [101]. Notably, the risk of bacterial infections was further enhanced if patients possessed both a medium-/high-risk haplotype of *FCN2* and a medium-/high-risk genotype of *MBL2* [101].

Following genotyping of 219 severely injured patients admitted to a level 1 trauma centre over a period of 3 years, a *FCN2* +6424G>T SNP was identified that predisposed patients to positive wound cultures and septic shock [102]. Additionally, this *FCN2* SNP, but not the -4A>G or -602G>A polymorphisms, was significantly associated with chronic adenotonsillitis in young children [103].

Although the vast majority of SNPs are associated with a negative output, three SNPs within *FCN2* have been associated with protection against pulmonary tuberculosis. In this study, the frequency of the -557A>G, -64A>C, and +6424G>T SNPs was found to be lower in the pulmonary TB group in comparison to the control group [104]. In opposition, research from Chalmers et al. suggests that there is no relationship between lectin pathway proteins and susceptibility to tuberculosis, leaving the subject open for debate [105]. Conversely, in a Chinese cohort of leprosy patients, genetic variants of *FCN2* (-557A>G and +6424G>T) in the promoter region and exon 8, respectively, were linked to low L-ficolin levels and had a positive association with leprosy susceptibility [106], which is in opposition to both H- and M-ficolin, where higher concentrations of either of these results in enhanced susceptibility [95, 107].

SNPs in *FCN2* have also been reported to be correlated with a predisposition to parasitic infections, including, leishmaniasis, malaria, Chagas disease, and schistosomiasis [108–111].

Elevated levels of L-ficolin observed in leishmaniasis were reported to be due to the +6359C>T structural variant [108]. Conversely, low L-ficolin levels were significantly associated with Chagas disease, which could be attributed to increased incidence of the Ala258Ser amino acid change (+6424G>T in exon 8) [110]. Moreover, heterozygote -4A>G genotypes with the Ala258Ser variant were more frequent amongst the patients with cardiodigestive symptoms [110].

Malaria patients were tested for polymorphisms known to be associated with varying L-ficolin plasma levels, and, although concentrations varied between mild and severe cases of malaria, there was no significant association for any of the haplotypes with disease severity [109].

Ouf et al., [111] identified that the -986G>AA and -4A>G alleles were significantly associated with schistosomiasis. Patients heterozygous for -986G>A or -4A>G, or those with the haplotype AGGG (composed of -986/-602/-4/+6424), were most at risk for schistosomiasis, whereas those homozygous for these mutations, or patients with the haplotype GGAG, were protected against schistosomiasis. Notably, L-ficolin concentrations were higher in both the controls and the GGAG patients.

SNPs in *FCN2* have also been implicated in viral infections, participating in the pathophysiology of hepatitis B infection [112]. There appeared to be significant haplotypic differences between hepatitis B patients and controls, with the AGGG haplotype being found more frequently in controls and the AAAG haplotype being associated with higher L-ficolin concentrations (both composed of -986/-602/-4/+6424) and increased viral loads [112]. Furthermore, strong linkage was associated between the variant -986G>A and -4A>G.

### 7.3. SNPs in *FCN3*

As aforementioned, *FCN3* was observed to have fewer SNPs comparatively to *FCN1* and *FCN2*, and few have been implicated in pathogen susceptibility.

The first incidence of H-ficolin deficiency was highlighted by Munthe-Fog et al. in 2009 [113], whereby they identified a patient with recurrent infections that was homozygous for a frameshift mutation (+1637CdC) and had undetectable levels of H-ficolin in their serum. Furthermore, Michalski et al. [114] reported a neonate with *S. agalactiae* infection who was homozygous for this frameshift mutation and completely H-ficolin deficient, suggesting an important role of H-ficolin in antibacterial defence.

Recently, there has also been a link to leprosy. Although there was no direct link observed between polymorphisms in *FCN3* and leprosy, H-ficolin levels were higher in patients with the *FCN3* +4473C>A ^∗^2B1 haplotype, in addition to being higher in the leprosy and lepromatous patient's comparative to controls [107], indicating that elevated H-ficolin levels may help propagate the disease.

## 8. Conclusions

There has been ever increasing evidence that ficolins play an integral role in a plethora of infectious diseases, additionally mutations within these proteins generally results in enhanced susceptibility to infection. In recent years, there has been a lot of interest in the role of ficolins within fungal disease, with each of the human ficolins and rodent ficolin-A proving beneficial to antifungal immunity. Furthermore, the recognition spectra of both M- and H-ficolin are increasing, but compared to L-ficolin, are still relatively limited. Future research could therefore focus on further exploration of the role of M-ficolin within antifungal immunity and enhancing our knowledge on the roles of M- and H-ficolin within innate immunity.

## Figures and Tables

**Table 1:** Expression, sugar specificity, and target pathogens of human and rodent ficolins.

	Tissues of origin	Gene localization	Sugars	PAMPs	Endogenous/artificial ligands	Pathogen interactions
*Human*						
M-ficolin	Cell surface, serum	9q34	GlcNAc, GalNAc, LacNAc, SiaLacNAc, CysNAc, sialic acid, gangliosides	Ebola virus glycoprotein, chitin, *β*-1, 3-glucans	Acetylated human albumin CD43	*S. agalactiae*, *S. aureus*, *S. pneumoniae*, *S. mitis*, *E. coli*, IAV, *T. cruzi*, *Zaire Ebola virus*, *A. fumigatus*
L-ficolin	Serum	9q34	GlcNAc, GalNAc, ManNAc, CysNAc, GlyNAc, NeuNAc, acetylcholine, elastin	*β*-1, 3-glucans, N-glycans, HA, neuraminidase, teichoic acid, LPS	Acetylcholine, elastin, corticosteroids	*S. aureus*, *S. pyogenes*, *S. agalactiae*, *B. subtilis*, *S. typhimurium*, *E. coli*, *S. pneumoniae*, *L. monocytogenes*, *M. bovis* BCG, *M. tuberculosis*, *M. smegmatis*, *E. faecalis*, *A. fumigatus*, HCV, IAV, *T. cruzi*, *G. intestinalis*, *Leptospira biflexa*, *Pasteurella pneumotropica*
H-ficolin	Serum, bronchus, alveolus, bile	1p36.11	GlcNAc, GalNAc, fucose, glucose, acetylsalicylic acid, sialic acid, D-mannose, GlyNAc, CysNAc	LPS, PSA, Ag85	—	*S. typhimurium*, *S. minnesota*, *E. coli* O111, *Hafnia alvei*, *A. fumigatus*, IAV, *T. cruzi*, *G. intestinalis*, *P. pneumotropica*, *M. bovis* BCG, *M. kansasii*
*Rodent*						
Ficolin-A	Serum	2A3	GlcNAc, GalNAc	LPS	Fibrinogen	*S. pneumoniae*, *S. aureus*, *E. coli* O157:H7, *P. aeruginosa*, *C. neoformans*, *A. fumigatus*, *A. flavus*, *A. terreus*, *A. niger*
Ficolin-B	Peritoneal MØ	2A3	GlcNAc, GalNAc, LacNAc, SiaLacNAc, LDL, NeuNAc	—	LDL, fetuin	Nk

BM, bone marrow; GlcNAc, *N*-acetylglucosamine; GalNAc, *N*-acetylgalactosamine; LacNAc, *N*-acetyllactosamine; SiaLacNAc, sialylated *N-*acetyllactosamine; CysNAc, *N-*acetylcysteine; ManNAc, *N-*acetylmannosamine; GlyNAc, *N-*acetylglycine; NeuNAc, *N-*acetylneuraminic acid; HA, hemagglutinin; LPS, lipopolysaccharide; IAV, influenza A virus; LDL, low-density lipoprotein; LTA, lipoteichoic acid; HCV, hepatitis C virus; Nk, not known.

**Table 2:** Single nucleotide polymorphisms in ficolin genes contributing to pathophysiology and colonization.

Ficolin gene	rs number	Position	Gene region	Amino acid change	Reference
M-ficolin (*FCN1*)	rs2989727	-1981G>A	Promoter	—	[93, 94]
	rs10120023	-542G>A	Promoter	—	[95]
	rs10117466	-144C>A	Promoter	—	[95]
	rs148669884	+6658G>A	Exon 8	Ala218Thr	[93]
	rs150625869	+7895T>C	Exon 9	Ser268Pro	[93]
	rs1071583	+7918G>A	Exon 9	Gln275Gln	[94]
	rs138055828	+7959A>G	Exon 9	Asn289Ser	[93]

L-ficolin (*FCN2*)	rs3124952	-986G>A	Promoter	—	[111, 112]
	rs3124953	-602G>A	Promoter	—	[103]
	rs3811140	-557A>G	Promoter	—	[104, 106]
	rs28969369	-64A>C	Promoter	—	[96, 104]
	rs175141136	-4A>G	Promoter	—	[103, 111, 112]
	rs17549193	+6359C>T	Exon 8	Thr236Met	[96, 98–100, 108]
	rs7851696	+6424G>T	Exon 8	Ala258Met	[96, 97, 102, 104, 106, 110]

H-ficolin (*FCN3*)	rs28357092	+1637CdC	Exon 5	Leu117fs	[113, 114]
	rs4494157	+4473C>A	Intron 7	—	[107]
